# Spectral and Multifractal Signature of Cortical Spreading Depolarisation in Aged Rats

**DOI:** 10.3389/fphys.2018.01512

**Published:** 2018-11-08

**Authors:** Péter Makra, Ákos Menyhárt, Ferenc Bari, Eszter Farkas

**Affiliations:** Department of Medical Physics and Informatics, University of Szeged, Szeged, Hungary

**Keywords:** cortical spreading depolarisation, short-time Fourier transform, multifractal, detrended fluctuation analysis, local field potential

## Abstract

Cortical spreading depolarisation (CSD) is a transient disruption of ion balance that propagates along the cortex. It has been identified as an important factor in the progression of cerebral damage associated with stroke or traumatic brain injury. We analysed local field potential signals during CSD in old and young rats to look for age-related differences. We compared CSDs elicited under physiological conditions (baseline), during ischaemia and during reperfusion. We applied short-time Fourier transform and a windowed implementation of multifractal detrended fluctuation analysis to follow the electrophysiological signature of CSD. Both in the time-dependent spectral profiles and in the multifractal spectrum width, CSDs appeared as transient dips, which we described on the basis of their duration, depression and recovery slope and degree of drop and rise. The most significant age-related difference we found was in the depression slope, which was significantly more negative in the beta band and less negative in the delta band of old animals. In several parameters, we observed an attenuation-regeneration pattern in reaction to ischaemia and reperfusion, which was absent in the old age group. The age-related deviation from the pattern took two forms: the rise parameter did not show any attenuation in ischaemic conditions for old animals, whilst the depression slope in most frequency bands remained attenuated during reperfusion and did not regenerate in this age group. Though the multifractal spectrum width proved to be a reliable indicator of events like CSDs or ischaemia onset, we failed to find any case where it would add extra detail to the information provided by the Fourier description.

## 1. Introduction

Cortical spreading depolarisation or depression (CSD) is a self-propagating wave of depolarisation along the cortex (Leão, 1944; Somjen, 2001). In recent years, it has gained significance as it has been recognised as a key factor in the progression of secondary tissue damage after subarachnoid haemorrhage, stroke, or traumatic brain injury (Hartings et al., 2016). CSDs have also been put forward in neurocritical care as indicators of the degree of metabolic failure in the nervous tissue (Dreier et al., 2016). The influences that regulate the occurrence and propagation of CSDs have not yet been fully explored, which justifies further research.

A decisive factor that is under extensive study is aging. Age brings multiple changes to the biochemistry and cellular make-up of the cortex, and, in accordance with this, several age-related differences in CSD dynamics have come to light: for example, the speed of propagation is slower (Guedes et al., 1996) or the duration of CSDs is shorter in aged rats (Farkas et al., 2011). Also, the three subsequent elements of the CSD-related cerebral blood flow (CBF) response, namely an initial, transient drop in CBF, the subsequent marked hyperaemia, and the ensuing, long-lasting oligaemia were all found to be attenuated with aging as shown in rats (Farkas et al., 2011; Menyhárt et al., 2015). This observation is especially significant taken that an age-specific haemodynamic response to CSD has been proposed to play an important role in injury progression in the aged brain (Farkas and Bari, 2014).

One of the most direct and spectacular electrophysiological indicators of the onset of CSDs is the direct current (DC) potential, which is essentially the component of the local field potential (LFP) that remains after low-pass filtering. Most studies use the transient deflection in the DC potential as the electrophysiological signature of CSD and use its morphological parameters to characterise CSD evolution. Yet CSDs also cause the full-band LFP amplitude to drop, reflecting a period of highly attenuated function and a loss of excitability in the neural tissue, and making the full-band LFP as feasible a target in offline investigations as the DC potential.

Exploring the spectral fine structure of cortical electrophysiological signals in the established frequency bands (delta to gamma) may also contribute to our understanding of CSD dynamics. The alpha-to-delta ratio (ADR), for example, has proved to be a predictor of worse recovery from ischaemia in humans (Claassen et al., 2004). Following the time dependence of the spectral powers in the standard frequency ranges can yield some additional information on the evolution and age dependence of CSDs (Menyhárt et al., 2015; Hertelendy et al., 2016).

An emergent tool in the study of complex system dynamics is multifractal analysis. Heralded as a promising method to disentangle multi-scale interactions and phase transitions in complex systems, it has recently gained ground in several areas of biomedical research and psychology from the segmentation of medical images (Lopes and Betrouni, 2009) through neuroscience (Zheng et al., 2005; Ihlen and Vereijken, 2010; Zorick and Mandelkern, 2013) to even long-range correlations in narrative texts (Drożdż et al., 2016).

In this paper, we set out to explore how aging impacts the evolution of the spectral power of LFP during CSDs in distinct frequency bands (delta, theta, alpha and beta). Furthermore, we seek to incorporate multifractal analysis into the investigation of CSD dynamics to see if it reveals anything beyond the insights provided by the Fourier technique.

## 2. Materials and methods

### 2.1. Experimental protocol

The data we analyse in this paper originate from an earlier study reported, and all surgical and experimental procedures are, therefore, identical to those previously published (Menyhárt et al., 2017b). Briefly, the specimens were young adult (2 month-old, *n* = 20) and old (18–20 month-old, *n* = 18) male Sprague-Dawley rats. Our experimental procedures conformed to the guidelines of the Scientific Committee of Animal Experimentation of the Hungarian Academy of Sciences (updated Law and Regulations on Animal Protection: 40/2013. [II. 14.] Govt of Hungary), following the EU Directive 2010/63/EU on the protection of animals used for scientific purposes and were approved by the National Food Chain Safety and Animal Health Directorate of Csongrád County, Hungary.

**Figure 1:**
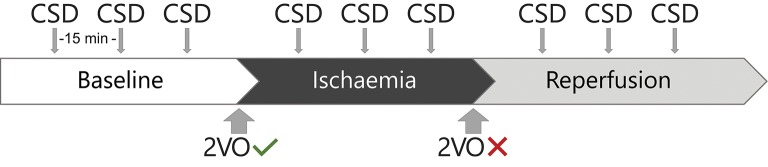
The experimental protocol. 2VO indicates two-vessel occlusion.

The animals were anaesthetised with isoflurane. After a baseline period lasting 50 min, we induced global forebrain ischaemia with the bilateral occlusion of the common carotid arteries (two-vessel occlusion, 2VO). An hour later, we released the carotid arteries to allow the reperfusion of the forebrain. Reperfusion also lasted for an hour. In all experimental stages (i.e., baseline, ischaemia, and reperfusion), we elicited three CSDs with the topical application of 1 M KCl at even intervals of 15 min (see Figure 1). Experiments were terminated by an overdose of isoflurane.

In the rat, the bilateral occlusion of the common carotid arteries is a widely accepted procedure to induce incomplete global forebrain ischaemia (Farkas et al., 2007). The time window for ischaemia (i.e., 1 h) in our experiments was chosen for a number of reasons, including: (i) Ischaemia is the most consistent during the first few hours after the occlusion of the carotid arteries; (ii) Ischaemia during this period of time is similar to the penumbra typically evolving in focal ischaemic stroke, a region highly relevant for medical intervention; (iii) One hour of ischaemia offers a long enough period to trigger 3 CSD events, 15–20 min apart—this number of CSD events is necessary to confirm reproducibility and reliability; the inter-CSD interval is necessary for the tissue to recover from each CSD before the next event is provoked; (iv) The duration of the surgical procedures and the experimental protocol (together reaching 10 h) does not allow much longer period of ischaemia monitoring, taken that the animals are terminated at the end of the experimental protocol for ethical and other practical reasons. The experimental protocol to induce ischaemia was published repeatedly in our previous papers, which justifies its validity (Hertelendy et al., 2016; Varga et al., 2016; Menyhárt et al., 2017a,b).

We monitored the local field potential (LFP) in the cortex with a glass capillary electrode through a cranial window, relative to an Ag/AgCl reference electrode implanted under the skin of the neck of the animal. The LFP signal was amplified, filtered, conditioned and finally digitised at a sampling frequency of 1 kHz by a setup identical to that described in Hertelendy et al. (2016) and Menyhárt et al. (2017b).

### 2.2. Spectral analysis

We carried out all signal analysis tasks (spectral and multifractal) in a self-developed .NET environment written in C#. Fast Fourier transforms were calculated using a .NET wrapper around FFTW (*the Fastest Fourier Transform in the West*, http://www.fftw.org/). We also leveraged the Task Parallel Library (TPL) included in .NET to speed up calculations.

#### 2.2.1. Artefact filtering

**Figure 2:**
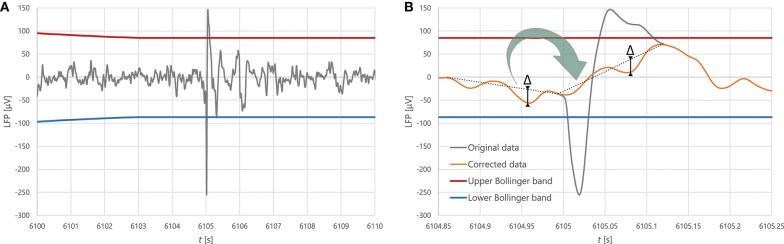
**(A)** A typical artefact. **(B)** The process of artefact correction: the segment classified as artefact is replaced by a transform of the preceding segment of equal length, where the slope of the line between the endpoints is changed whilst the distance between this line and other individual points of the data segment (labelled Δ in the figure) stays invariant in the process of transformation.

Before all further analysis, we filtered the local field potential (LFP), removing excessive spikes that had likely resulted from measurement artefacts, in order to prevent them from contaminating the spectrum. To avoid subjectivity in deciding what constitutes an artefact, we calculated the Bollinger bands (moving average ± moving standard deviation) in a window of 10,000 points, and marked segments where the magnitude of the signal exceeded a threshold of the local mean plus 4.2 times the local standard deviation as potential artefacts. We reviewed each automatic detection and only considered a segment as artefact if its slope was uncharacteristically steep. We removed confirmed artefacts and replaced them with a slope-corrected copy of the preceding interval of equal length. We applied cubic spline interpolation in a 5-point radius of the junctions between the original signal and the correction to ensure that the signal stays smooth. We found no more than 10 artefacts on average per CSD event, which altogether lasted <1 s, about 0.1–0.15% of the duration of a CSD event. The process is illustrated in Figure 2.

#### 2.2.2. Short-time fourier transform (STFT)

The basis of the spectral investigations is the short-time Fourier transform (STFT) of the relevant LFP sequence ${\left\{x_{k}\right\}}_{k=0}^{N-1}.$ Advancing a Gaussian window *w*_*k*_ of width *W*Δ*t* = 60s (wherein Δ*t* = 0.001s denotes the sampling interval) along the LFP sequence in steps of Δτ = 1s, one can obtain the STFT value at time *t*_*m*_ = *m*Δ*τ* and frequency $f_{n}=n\Delta f=\frac{n}{W\Delta t}$ as

(1) $$\begin{array}{l} X\left(t_{m},f_{n}\right)=X_{m,n}=\overset{W-1}{\underset{k=0}{\sum }}w_{k}x_{m+k}{\text{e}}^{-\text{i}\frac{2\pi }{N}kn}. \end{array}$$

From the STFT, a time-dependent power spectral density *S*(*t*_*m*_, *f*_*n*_) can be calculated as follows:

(2) $$\begin{array}{l} S\left(t_{m},f_{n}\right)=S_{m,n}=\frac{|X_{m,n}{|}^{2}\Delta t}{W}. \end{array}$$

#### 2.2.3. Spectral power

The integrated spectral power *P*(*t*_*m*_) = *P*_*m*_ of a given frequency range between *f*_min_ = *n*_min_Δ*f* and *f*_max_ = *n*_max_Δ*f* is the integral of the power spectral density between these limits, which, in discrete representation can be calculated as

(3) $$\begin{array}{l} P\left(t_{m}\right)=P_{m}=\overset{n_{\text{max}}-1}{\underset{n=n_{\text{min}}}{\sum }}S_{m,n}\Delta f=\overset{n_{\text{max}}-1}{\underset{n=n_{\text{min}}}{\sum }}\frac{|X_{m,n}{|}^{2}\Delta t}{W}\cdot \frac{1}{W\Delta t}\\ =\overset{n_{\text{max}}-1}{\underset{n=n_{\text{min}}}{\sum }}\frac{|X_{m,n}{|}^{2}}{W^{2}}. \end{array}$$

The four frequency ranges of brain electrical activity defined in Table 1 were analysed.

**Table 1:** The frequency ranges used in the analysis.

**Frequency range**	**Minimum frequency [Hz]**	**Maximum frequency [Hz]**
Alpha	8	13
Beta	13	30
Delta	1	3
Theta	3	8

### 2.3. Multifractal detrended fluctuation analysis (MFDFA)

In addition to the Fourier spectrum, we also applied multifractal detrended fluctuation analysis (MFDFA) to our LFP sequences. We followed the procedure laid out in Kantelhardt et al. (2002) and Ihlen (2012). For a given interval ${\left\{x_{k}\right\}}_{k=0}^{N-1}$ of the LFP, we first constructed a cumulative sum *Y* of the data series:

(4) $$\begin{array}{l} Y_{i}=\overset{i}{\underset{k=0}{\sum }}x_{k}-\langle x\rangle \;\left(0\leq i<N\right), \end{array}$$

wherein 〈*x*〉 denotes the mean of the interval. Then we partitioned the cumulative sum sequence *Y* into disjunct segments of equal length *s*. Since the length *N* of the interval is usually not an integer multiple of the segment size *s*, we repeated the partitioning process from the opposite end, thus obtaining 2*N*_*s*_ segments altogether, where $N_{s}=\left\lfloor \frac{N}{s}\right\rfloor$ is the number of segments in a single direction (⌊…⌋ denotes rounding down). Detrending meant the subtraction of a local polynomial trend *y*_ν_, after which we calculated the local variance as

(5) $$\begin{array}{l} F_{\nu }^{2}\left(s\right)=\frac{1}{s}\overset{s-1}{\underset{i=0}{\sum }}{\left\{Y_{\nu s+i}-y_{\nu ,i}\right\}}^{2} \end{array}$$

in the forward direction (0 ≤ ν < *N*_*s*_), and as

(6) $$\begin{array}{l} F_{\nu }^{2}\left(s\right)=\frac{1}{s}\overset{s-1}{\underset{i=0}{\sum }}{\left\{Y_{N-\left(\nu -N_{s}+1\right)s+i}-y_{\nu ,i}\right\}}^{2} \end{array}$$

in the reverse direction (*N*_*s*_ ≤ ν < 2*N*_*s*_), where *y*_ν_ is the local trend for the νth segment, obtained using *m*-order polynomial least squares fitting. The central quantity of MFDFA is the *q*th-order fluctuation function defined as

(7) $$\begin{array}{ll} F\left(s,q\right) & ={\left\{\frac{1}{2N_{s}}\overset{2N_{s}-1}{\underset{\nu =0}{\sum }}{\left[F_{\nu }^{2}\left(s\right)\right]}^{q/2}\right\}}^{1/q}\;\left(q\neq 0\right), \end{array}$$

(8) $$\begin{array}{ll} F\left(s,0\right) & =\exp\left\{\frac{1}{4N_{s}}\overset{2N_{s}-1}{\underset{\nu =0}{\sum }}\ln\left[F_{\nu }^{2}\left(s\right)\right]\right\}\;\left(q=0\right). \end{array}$$

The fractal properties of our time series *x* are reflected in how this fluctuation function depends on the scale *s*, whilst the multifractal order *q* serves to level the contributions made by components of small effective value and by those of large effective value: negative values of *q* amplify processes of small fluctuations and positive values enhance large fluctuations. The value *q* = 2 corresponds to standard monofractal analysis, wherein the effective value of the local variance is calculated as a function of the segment size *s*. We obtained the generalised Hurst exponent *h*(*q*) from the slope of the ln[*F*(*s, q*)] vs. ln(*s*) graph using linear least-squares regression. As described in Ihlen (2012), Eke et al. (2002), and Eke et al. (2000), this slope yields the Hurst exponent *h*(*q*) directly for a class of signals called fractional Gaussian noise (fGn), whereas for another class, fractional Brownian motion (fBm), the slope equals 1 + *h*(*q*). Our preliminary classification showed that our LFP signals fall into the latter category (as most physiological signals do), so we subtracted one from the slope. Finally, we calculated the singularity strength as

(9) $$\begin{array}{l} \alpha =h\left(q\right)+q\frac{\text{d}h}{\text{d}q}, \end{array}$$

and the singularity dimension as

(10) $$\begin{array}{l} f\left(\alpha \right)=q\left[\alpha -h\left(q\right)\right]+1. \end{array}$$

**Figure 3:**
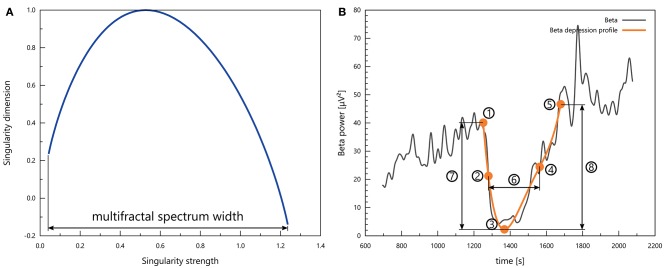
**(A)** A representative multifractal spectrum with the definition of multifractal spectrum width. **(B)** Characteristic points and measures of a depression profile: 1 – baseline; 2 – mid-depression; 3 – depression; 4 – mid-recovery; 5 – recovery; 6 – depression duration; 7 – drop; 8 – rise.

What we called the multifractal spectrum was the *f*(α) function as a parametric curve that depends on the multifractal order *q*. We focused on the multifractal spectrum width Δα (see Figure 3A), defined as

(11) $$\begin{array}{l} \Delta \alpha =\alpha_{\text{max}}-\alpha_{\text{min}}. \end{array}$$

In a similar way to STFT, we applied a windowed implementation of MFDFA: we calculated the multifractal properties in a 60-s window, then advanced the window by a step of 1 s and repeated the process, obtaining time-dependent functions comparable to the spectral powers discussed above.

In our investigations, the segment size varied from 16 to 512 as powers of 2. The maximum segment size was constrained by the requirement of scale invariance discussed in Ihlen (2012), and we decided upon a detrending order *m* = 1 as this choice yielded the widest range of approximate scale invariance. We varied the multifractal order *q* between −5 and 5 in 100 even steps. We tested the reliability of our calculations on white noise sequences and on the binomial multifractal series described in Kantelhardt et al. (2002).

### 2.4. Depression profiles

Cortical spreading depolarisation events appeared as periods of transient drop in all spectral powers and in the multifractal spectrum width. To quantify the properties of these intervals, we searched for the best 4th-order polynomial fit for the depression in the signal. The initial candidates for the beginning and the end of such depression intervals we selected manually, but then an automatic algorithm could override these if it found a better fit with an endpoint within 10 s of the initial estimate. Polynomials which showed non-monotonicity in the neighbourhood of the endpoints were rejected. We designated five characteristic points to describe depression profiles (see Figure 3B):

- Baseline point, whose *y* value is the mean of the respective signal in a 60-s interval before the onset of the depression and whose time coordinate is the time instant at which the best polynomial fit intersects this constant level;
- Depression point, which is simply the minimum of the polynomial in the depression profile;
- Mid-depression point, where the polynomial takes on a value equal to the arithmetic mean of the baseline value and the depression value;
- Recovery point, whose *y* value is the mean of the respective signal in a 60-s interval after the end of the depression and whose time coordinate is the time instant at which the best polynomial fit intersects this constant level; and
- Mid-recovery point, where the polynomial assumes a value equal to the arithmetic mean of the depression value and the recovery value.

We standardised each depression profile by subtracting the mean and dividing by the standard deviation, where the mean and the standard deviation were calculated for a signal segment that lasted from the end of the previous CSD to the beginning of the next. We evaluated the following quantifiers for a standardised depression profile (see Figure 3B):

- Depression duration—the time that passes from mid-depression to mid-recovery;
- Depression slope—the derivative of the polynomial fit at the mid-depression point (divided by the standard deviation as discussed above);
- Drop—the difference between the standardised baseline value and the standardised depression value;
- Recovery slope—the derivative of the polynomial fit at the mid-recovery point (divided by the standard deviation as discussed above); and
- Rise—the difference between the standardised recovery value and the standardised depression value.

### 2.5. Statistics

We used R for all our statistical calculations. Except where otherwise noted, we divided our data into six subgroups according to age (young or old) and experimental stage (baseline, ischaemia, and reperfusion). We did not include the first CSD in the baseline group as it represents a markedly different physiological state to all subsequent CSDs, even those in the baseline group. In each experimental group, we applied a Grubbs test to decide whether extreme values are outliers. Proven outliers were removed. Then we used two-way ANOVA with age and experimental stage as factors, followed by Tukey's honest significant differences (HSD) as a *post-hoc* test to obtain pairwise comparisons. In the figures and the text, data are given as mean±standard error of the mean.

## 3. Results

**Figure 4:**
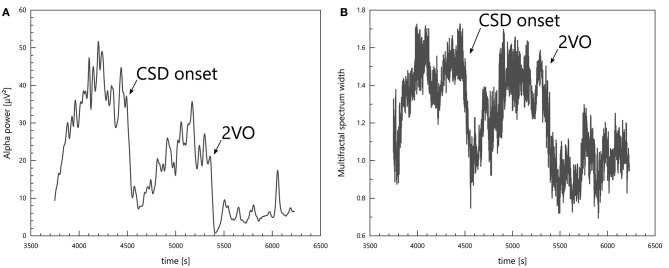
Signs of a CSD and of ischaemia induction (2VO) in the alpha power **(A)** and in the multifractal spectrum width **(B)**.

Changes in the physiological state of the specimens were reflected unambiguously in the spectral power in all bands and also in multifractal spectrum width. CSDs and ischaemia induction (2VO) caused both spectral powers and multifractal spectrum width to drop (see Figure 4).

### 3.1. Duration of depression

**Figure 5:**
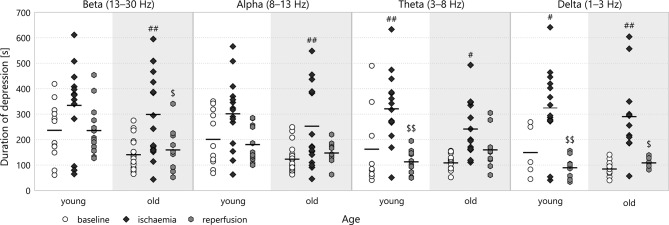
Duration of depression in different frequency bands for all experimental stages. Significance levels are given as ^#^*p* < 0.05, ^*##*^*p* < 0.01 v respective *baseline;* ^*$*^*p* < 0.05, ^*$$*^*p* < 0.01 v respective *ischaemia*.

**Figure 6:**
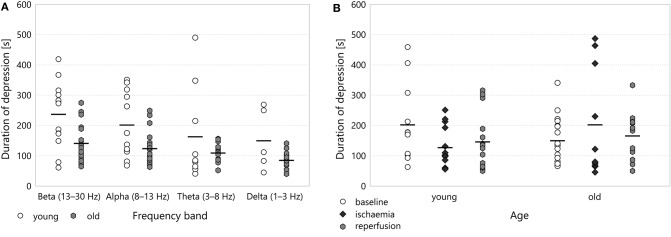
**(A)** Duration of depression in different frequency bands for the baseline period. A two-way ANOVA with age and frequency band as factors showed significant difference with respect to age (*p* = 0.00089) overall, though Tukey's HSD applied as a *post-hoc* test did not find significant pairwise difference between any two comparable age– band groups. **(B)** Duration of depression in the multifractal spectrum width.

Ischaemia lengthened the LFP depression in all frequency bands (see Figure 5). This effect was most significant in the theta band (e.g., 323.47 ± 35.32 s for *young*–*ischaemic* v 165 ± 52.19 s for *young*–*baseline*, *p* = 0.0058). The multifractal spectrum width did not show such a discernible pattern: for old animals, the depression tended to be longer during ischaemia, but for young ones, the relation was the opposite (see Figure 6B). These apparent differences in the multifractal spectrum width did not prove significant, however.

Though the duration of depression was clearly shorter in old animals throughout all experimental stages (most visibly in the baseline stage), this categorisation did not show any statistically significant difference according to age. To reveal potential aging effects, we run another two-way ANOVA restricted to the baseline stage, this time with age and frequency band as its factors (see Figure 6A). This test indicated a significant difference according to age overall (192.14 ± 10.68 s for *old* v 246.63 ± 12.31 s for *young*, *p* = 0.00089) but Tukey's HSD did not find any significant difference in the pairwise comparisons between individual groups.

### 3.2. Depression slope

**Figure 7:**
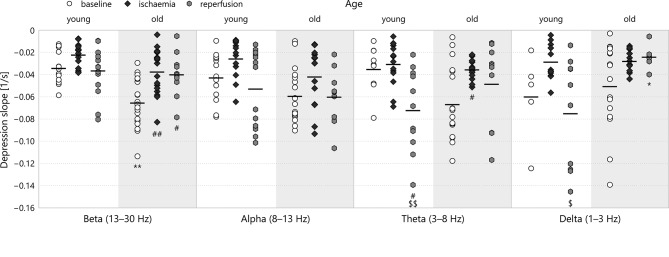
Depression slope in the different frequency bands of LFP power. Significance levels are given as ^*^p < 0.05, ^**^p < 0.01 v respective *young*; ^#^*p* < 0.05, ^*##*^*p* < 0.01 v respective *baseline;* ^*$*^*p* < 0.05, ^*$$*^*p* < 0.01 v respective *ischaemia*.

Of all the parameters we investigated, the slope of depression showed the effects of aging the most clearly (see Figure 7). In the beta, alpha and theta bands, old age resulted in a steeper decrease of the spectral power in the baseline stage. This was markedly significant for the beta band (−0.065 ± 0.005s^−1^ for *old*–*baseline* v −0.034 ± 0.004s^−1^ for *young*–*baseline*, *p* = 0.00026). In all spectral bands, ischaemia reduced the absolute value of the depression slope, most significantly again in the beta band (−0.037 ± 0.005s^−1^ for *old*–*ischaemia* v −0.065 ± 0.005s^−1^ for *old*–*baseline*, *p* = 0.00045). For young animals, reperfusion restored the slope of depression or made it even steeper in the lower frequency bands theta and delta (in the theta band: −0.072 ± 0.011s^−1^ for *young*–*reperfusion* v −0.035 ± 0.007s^−1^ for *young*–*baseline*, *p* = 0.049). This regeneration of slope also occurred in old animals in the middle frequency bands alpha and theta (in the alpha band: −0.060 ± 0.009s^−1^ for *old*–*reperfusion* v −0.042 ± 0.006s^−1^ for *old*–*ischaemia*, *p* = 0.51), but was clearly absent from the beta and delta bands. Again, the multifractal spectrum width did not show any significant difference according to age or experimental stage.

### 3.3. Drop

**Figure 8:**
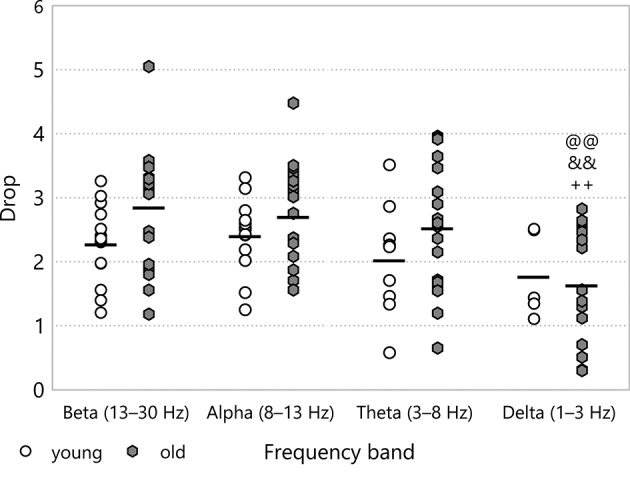
Drop during baseline CSDs in the different frequency bands of the LFP power. Significance levels were obtained using two-way ANOVA with age and frequency band as factors, with Tukey's HSD as a *post-hoc* test, and are given as ^@@^ *p* < 0.01 v respective *beta*; ^&&^ *p* < 0.01 v respective *alpha* and ^++^ *p* < 0.01 v respective *theta*.

**Figure 9:**
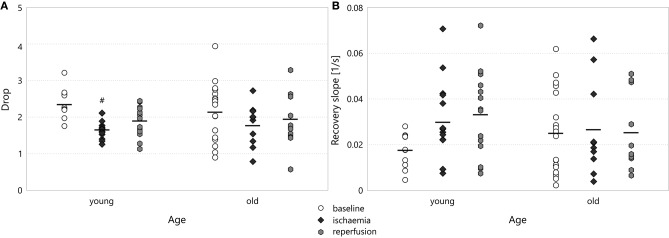
**(A)** Drop in the multifractal spectrum width. Significance levels are given as ^#^*p* < 0.05 v respective *baseline*. **(B)** Recovery slope in the multifractal spectrum width.

The drop in the spectral power did not show any significant difference between experimental groups. When we focused on the frequency bands in the baseline stage, however, we could discern some frequency dependence (see Figure 8). The drop was less at low frequencies, especially in the old age group (1.65 ± 0.22 for *old*–*delta* v 2.86 ± 0.21 for *old*–*beta*, *p* = 2.4·10^−5^). In the multifractal spectrum width, one could observe a smaller drop during ischaemia than in the baseline state, which was significant for young animals (1.67 ± 0.07 for *young*–*ischaemia* v 2.36 ± 0.12 for *young*–*baseline*, *p* = 0.048, see Figure 9A). This was the only case where the multifractal spectrum width proved significantly different to that in any other experimental group.

### 3.4. Recovery slope

**Figure 10:**
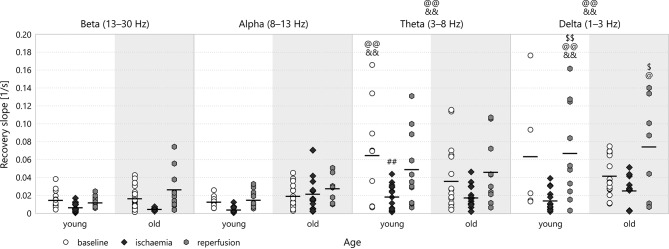
Recovery slope the different frequency bands of the LFP power. Significance levels were obtained using three-way ANOVA with age, experimental stage and frequency band as factors, with Tukey's HSD as a *post-hoc* test, and are given as ^*##*^*p* < 0.01 v respective *baseline*; ^*$*^*p* < 0.05, ^*$$*^*p* < 0.01 v respective *ischaemia*; ^@^*p* < 0.05, ^@@^*p* < 0.01 v respective *beta*; ^&&^*p* < 0.01 v respective *alpha*.

The recovery slope followed the same attenuation-regeneration pattern as the depression slope in young animals, though this effect proved significant only in the theta (0.019 ± 0.004s^−1^ for *young*–*ischaemia* v 0.065 ± 0.019s^−1^ for *young*–*baseline*, *p* = 0.0080, see Figure 10) and delta bands. For this parameter, however, the regeneration brought about by reperfusion persisted in the old age group in all spectral bands except the alpha. One can also observe that the recovery slope is markedly higher in the lower bands theta and delta than in the higher bands beta and alpha (e.g., 0.068 ± 0.017s^−1^ for *delta*–*young*–*reperfusion* v 0.012 ± 0.002s^−1^ for *beta*–*young*–*reperfusion*, *p* = 0.0002). As Figure 9B shows, the attenuation-regeneration pattern was absent from the multifractal spectrum width.

### 3.5. Rise

**Figure 11:**
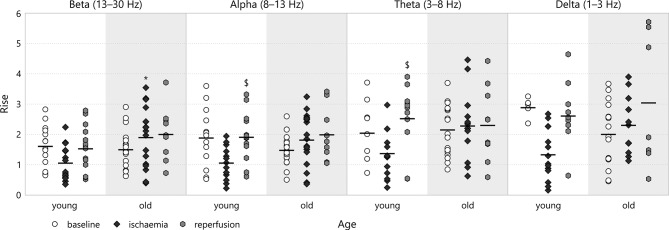
Rise in the different frequency bands of the LFP power. Significance levels are given as ^*^p < 0.05 v respective *young*; ^*$*^*p* < 0.05 v respective *ischaemia*.

A similar attenuation-regeneration dynamics also seemed to manifest itself in the rise after the CSD-induced transient depression for young animals (e.g., in the alpha band: 1.93 ± 0.22 for *young*–*reperfusion* v 1.08 ± 0.14 for *young*–*ischaemia*, *p* = 0.046; see Figure 11). This was clearly absent from the old age group as the rise remained at about the same level during ischaemia and reperfusion (e.g., in the beta band: 2.02 ± 0.26 for *old*–*reperfusion* and 1.92 ± 0.24 for *old*–*ischaemia*, the latter v 1.07 ± 0.15 for *young*–*ischaemia*, *p* = 0.029). We could observe no difference whatsoever in the multifractal spectrum width between experimental groups.

## 4. Discussion

Intraoperative electrocorticogram (ECoG) monitoring is an invasive approach to aid tumour resection or surgery for the alleviation of epilepsy (Yang et al., 2014; Alcaraz and Manninen, 2017), and it has lately been used for the post-operative monitoring of acute brain injury patients to follow the evolution of CSD events (Dreier et al., 2016). Although under most circumstances, full-band ECoG is sufficient to provide feedback to the neuro-surgeon or neuro-intensive care specialist, several studies have underpinned the clinical relevance of the analysis of brain electrophysiological signals by frequency band: in addition to alpha-to-delta ratio (Claassen et al., 2004), focal reduction in the alpha band of the electroencephalogram (EEG) can be associated with the occurrence of delayed cerebral infarction in subarachnoid haemorrhage (Gollwitzer et al., 2015); the slope of the EEG delta power correlates with stroke severity (Finnigan et al., 2004; Hartings et al., 2005); low beta power in the ECoG indicates a higher probability of CSD occurrence in patients with traumatic brain injury (Hertle et al., 2016) or the slope of theta power decline in EEG is a good predictor of postinjury epilepsy (Milikovsky et al., 2017).

Though age-associated changes have been revealed in the spectral composition of ECoG in rats–whilst aging causes high-frequency power to decrease, it also enhances delta power (Bagetta et al., 1989),–and on the other hand, the traces of CSD in scalp EEG frequency bands have been analysed (Hartings et al., 2014), the spectral implications of CSD have been mapped out in the EEG of conscious rabbits (Roshchina et al., 2014) and of conscious and anaesthetised rats (Koroleva et al., 2016), we have had no information on the influences of aging on the spectral representation of LFP during CSDs in different frequency bands. Since the pattern of CSD is considerably influenced by age (i.e., lower frequency of occurrence in the intact and ischaemic cortex, longer duration, and more frequent association with inverse CBF response in focal ischaemia) (Farkas et al., 2011; Clark et al., 2014; Menyhárt et al., 2015), here we set out to explore whether the analysis of LFP by frequency band or multifractal analysis might deliver any potential LFP signature specific for age. We presented LFP spectral analysis of CSD in a preliminary form earlier, but the age range covered in our previous report spanned young adulthood (7–30 weeks of age in rats), and did not go beyond to include old age as well (Hertelendy et al., 2016).

What we have found extends on the power spectrum-related conclusions in our earlier report (Hertelendy et al., 2016). There we observed a shorter duration of CSD-associated depression in the lower frequency bands delta and theta in 30-week-old animals and argued that owing to a low-pass filtering effect present in the brain tissue (Buzsáki et al., 2012), the distance from which lower-frequency components can reach the electrode is greater than that for higher frequencies, so a reduced duration in the lower frequency bands of older animals might indicate that at more distant sites, regeneration has already taken place, that is, the CSD-related depression wave is narrower in space for the 30-week-old group. Here, for 72–80-week-old animals, the duration of depression is shorter even in the high frequency bands alpha and beta, which, using the same logic, can mean that as age advances further, the CSD-related depression wave shrinks in space even more, to the point where its spatial extension does not exceed the range within which the electrode can detect high-frequency signals.

The most significant difference we found between young and old animals appeared in the depression slope of spectral powers after the onset of CSD. Under physiological conditions, CSD-induced decline in the beta power was much steeper in the old age group, whilst during reperfusion, the rate at which delta power decreased in reaction to CSDs was less in absolute value in old animals. The latter might be in accord with earlier findings where less negative or even positive delta slope (termed aDCI, acute delta change index) predicted worse outcomes in ischaemic stroke patients (Finnigan et al., 2004).

### 4.1. Attenuation-regeneration pattern

Several parameters investigated here followed a pattern where values decreased during ischaemia as compared to baseline then were restored during reperfusion. This behaviour was most consistent in the recovery slope. These data are consistent with the profound differences in the pattern of CSDs that occur in the intact and ischaemic cerebral cortex. As such, CSD as indicated by the negative deflection of the DC potential lasts significantly longer under ischaemia as compared with the intact condition (Menyhárt et al., 2015), and the coupled hyperaemia is of substantially smaller amplitude but longer duration (Menyhárt et al., 2017b). The slower LFP recovery from CSD under ischaemia, found here especially in the low frequency bands, faithfully reflects the lack of metabolic resources to re-establish resting electrical activity of the nervous tissue.

Most age-related effects we found represented a deviation from this pattern. The attenuation of the depression slope of beta and delta spectral powers proved permanent in old animals and was not followed by regeneration. Another type of age-related deviation could be observed in the rise after CSD-induced depressions in the spectral powers: here the attenuation step was absent from the spectrum of old animals and the rise values remained about the same throughout all experimental stages. Finally, we noted an isolated departure from attenuation-regeneration dynamics in the recovery slope of the alpha power of old animals, where again no attenuation occurred during ischaemia.

### 4.2. Multifractal spectrum to complement the fourier spectrum

One argument for the application of fractal analysis instead of or in addition to traditional linear investigation methods such as Fourier transform is the perceived inability of the latter to quantify the scale-dependent properties of complex biological systems that stem from the interplay of many levels of substructure (Chakraborty et al., 2016). In human EEG, the Fourier transform failed to show a response to olfactory stimuli, whilst the Hausdorff–Besicovitch fractal dimension proved sensitive to them (Murali and Vladimir, 2007). The fractal dimension increases after brain injury in rats (Spasic et al., 2005). The fractal dimension and the Hurst exponent differ before and after CSD (Santos et al., 2014) and monofractal detrended fluctuation analysis detected slight variations in the Hurst exponent before, during and after CSD (do Nascimento et al., 2010).

In addition to the monofractal studies above, several multifractal analyses have targeted the brain. The multifractal spectrum width calculated for the EEG database of epilepsy patients has been shown to be less in ictal periods than in interictal ones (Zhang et al., 2015), which was in agreement with the comparison of normal and epileptic rat EEG (Dutta, 2010).

To our knowledge, this paper is the first to extend earlier monofractal studies (do Nascimento et al., 2010; Santos et al., 2014) towards multifractality in the investigation of CSD. We demonstrated that CSDs cause a transient narrowing in the multifractal spectrum, signalling a temporary loss of multifractality and thus a suppression of the interplay between different scales in the LFP. This is in agreement with previous MFDFA-based findings: just like epilepsy (Dutta, 2010; Zhang et al., 2015), CSD is reflected in the multifractal spectrum as a reduction in width.

One goal of ours was to find out whether multifractal analysis yields any additional information on the dynamics of the local field potential during CSDs as compared to the Fourier spectrum. The MFDFA parameter we chose to follow in this study, the multifractal spectrum width, has failed to reveal anything more than our STFT-based profiles–in fact, it has proved largely insensitive to age or experimental stage. The only exception to this was the drop in the profile as a reaction to CSD, which, in young animals, was significantly less during ischaemia than the baseline, but it did not fit into any discernible pattern.

## Author contributions

PM: analysis and interpretation of data, drafting the article; ÁM: substantial contributions to conception and design, acquisition of data; FB: revising the manuscript critically for important intellectual content; EF: substantial contributions to conception and design, analysis and interpretation of data, drafting the article, and revising it critically for important intellectual content.

### Conflict of interest statement

The authors declare that the research was conducted in the absence of any commercial or financial relationships that could be construed as a potential conflict of interest.
